# Understanding the resurgence of mpox: key drivers and lessons from recent outbreaks in Africa

**DOI:** 10.1186/s41182-024-00678-1

**Published:** 2025-04-03

**Authors:** Adewunmi Akingbola, Christopher Abiodun Adegbesan, Olajumoke Adewole, Courage Idahor, Tolani Odukoya, Emmanuel Nwaeze, Shekoni Mayowa, Owolabi Abdullahi, Petra Kerubo Mariaria

**Affiliations:** 1 Department of Public Health, University of Cambridge, Cambridgeshire Old Trinity Schools, Cambridge, CB2 1TN England, UK; 2 Department of Global Health, African Cancer Institute, Stellenbosch University, Tygerberg, Cape Town, South Africa; 3 Department of Community Health and Primary Healthcare, Lagos State University College of Medicine, Ikeja Lagos, Nigeria; 4 Nottingham University Hospitals NHS Trust, Nottingham, UK; 5 School of Public Health, University of Port-Harcourt, Port-Harcourt, Rivers State Nigeria; 6 Department of Community Health, Lagos State University College of Medicine, Ikeja Lagos, Nigeria; 7 Department of Community Health and Primary Healthcare, Lagos State University College of Medicine, Ikeja Lagos, Nigeria; 8 Bayero University, Gwarzo Road, Gwale, PMB 3011, Kano, Kano State Nigeria; 9 Infectious Diseases Institute, Makerere University, Makerere, Uganda

**Keywords:** Mpox, Zoonoses, Disease outbreaks, Public Health Surveillance, Epidemiology, Viral zoonotic diseases

## Abstract

Mpox has re-emerged as a significant public health threat, particularly in Africa. This study explores the key drivers behind the recent resurgence, focusing on epidemiological trends, transmission dynamics, and lessons learned from recent outbreaks. The research involved a comprehensive review of recent mpox outbreaks, analyzing factors, such as socio-economic conditions, environmental influences, and genetic evolution. Findings indicate that the resurgence is linked to the cessation of smallpox vaccination, increased human–wildlife interactions, and rapid urbanization in endemic regions. The virus, which was previously confined to rural areas, has now spread to urban populations, and crossed national borders, driven by high population mobility and socio-economic instability. A notable shift in transmission dynamics has been observed, with increased human-to-human transmission, particularly among men who have sex with men (MSM), contributing to more severe and widespread outbreaks. The study highlights the urgent need to strengthen health systems in Africa, particularly in the areas of diagnostics, surveillance, and public health preparedness. Challenges such as inadequate laboratory infrastructure and delayed response mechanisms have exposed vulnerabilities in current public health frameworks. To prevent future outbreaks, targeted strategies must be implemented, including enhanced community engagement, improved access to vaccines and treatments, and timely, accurate reporting of cases. Coordinated global action is essential to prevent mpox from becoming a more persistent and widespread public health threat. This research discusses the importance of proactive measures and international cooperation in addressing the ongoing threat posed by mpox, particularly in regions with limited healthcare resources.

## Introduction

Human mpox is a zoonotic disease with a similar presentation as smallpox, caused by mpox virus, an enveloped double-stranded DNA virus belonging to the Poxiviridae family, Chordopoxviridae subfamily, and Orthopoxvirus genus [1, 2]. The mpox virus has two distinct clades; Clade 1 and Clade 2. Clade 1, previously known as the Central African Clade, has a higher virulence and case–fatality rate than Clade 2 (West African Clade) [3, 4]. Transmission of the disease can occur through animal–human contact (primary zoonotic transmission) via bite/scratch, close contact with infected animals, and by handling or consuming inadequately cooked meat of infected animals or human–human via the respiratory route or dermal route [5–9]. Mother–child transmission resulting in congenital mpox has also been reported [10]. Recent evidence has also identified sexual contact as a mode of transmission particularly among men who sleep with men [11]. The disease presents with clinical features similar to smallpox, with an incubation period ranging from 5 to 21 days, typically 6–13 days. An initial prodromal phase is characterized by fever, generalized headache, fatigue, and myalgia. There is also the presence of unilateral/bilateral cervical, axillary, or inguinal lymphadenopathy which appears with the onset of fever. This is followed by the appearance of maculopapular rash which frequently involves the palms and soles. The illness is self-limiting and resolves within 14–28 days. However, complications such as secondary infections, encephalitis, and blindness due to corneal involvement can occur [12, 13]. Pain management, treating the rash and preventing complications is the major goal of mpox treatment. Although there are no confirmed antiviral medications for treating mpox, antiviral medications like tecovirimat have been urgently authorized for use in some countries and are under evaluation in clinical trials [14]. There is an available vaccine for mpox, the JYNNEOS vaccine, which is recommended for individuals, like healthcare workers and close contacts of people with mpox, who have a high risk of contracting the disease, particularly during an outbreak. The vaccine can also be administered as a post exposure prophylaxis within 4 days in persons who have come in contact with individuals with mpox [15]. The virus was first isolated from monkeys kept in a research facility in Denmark in 1958, and the first human case was discovered in the Democratic Republic of the Congo in a 9-month-old child not vaccinated for smallpox, in 1970 [2, 16]. Twenty years after the discovery of the first case, over 400 cases were documented in Africa, with a vast majority of these cases diagnosed in the Democratic Republic of the Congo. Not too long after that, six new cases of infection with mpox virus were identified in some West African Countries, which include Nigeria, Liberia, and Sierra Leone [17, 18]. The disease has become endemic in Western and Central Africa, causing sporadic infections and outbreaks. In recent years, cases outside of Africa have emerged [19–22]. In the first half of 2022, over 3,000 laboratory cases and one death were reported to WHO from 5 WHO regions, with more than 80% of these cases reported from the WHO European region, and the mortality from Nigeria. Other affected regions included the region of the Americas, the Eastern Mediterranean Region, and the Western Pacific region [23]. This escalation resulted in WHO declaring the mpox outbreak a Public Health Emergency of International Concern (PHEIC) in July 2022. It ended the declaration in May 2023 after the cases were brought under control [24, 25]. However, in 2024, a significant increase in cases and deaths was recorded in the African region [26]. The Clade 2 virus was responsible for the outbreak which occurred in 2022. In contrast, multiple clades are responsible for the ongoing outbreak, particularly the more virulent and lethal clade 1b virus. The clade 1b virus was previously confined within the Democratic Republic of Congo but is now responsible for over 2000 cases and about 13 deaths in at least 13 African countries including countries surrounding the Democratic Republic of the Congo that have not recorded mpox cases before [24, 27–30]. Consequently, mpox disease was again declared a Public Health Emergency of International Concern (PHEIC), first by the African Center for Disease Control and Prevention (Africa CDC) on the 13th of August 2024, after which the WHO made a similar declaration the day after, just 15 months after the last outbreak was declared to be over [24, 30]. In contrast to previous outbreaks, evidence suggests that the spread of the mpox virus in the more recent outbreaks is linked to sexual contact, particularly among men who sleep with men [11]. In addition, compared to previous outbreaks that were largely confined to rural communities, this current outbreak is affecting urban areas with dense populations due to high population migration that has occurred over the years in which more people migrated from the rural to urban settlement for greener pastures, and better quality of life [28]. These factors increase the risk of the transcontinental spread of the virus which has the potential to lead to another global pandemic. This study seeks to explore the epidemiological trends and transmission dynamics of the mpox virus in Africa, identify drivers of the resurgence as well as highlight lessons from the recent outbreak within the continent, and provide future directions and recommendations that will help combat this epidemic and mitigate a possible pandemic.

### Epidemiological trends and transmission dynamics

Mpox virus, a member of the genus Orthopoxvirus, of the family Poxviridae, is the causative agent of mpox in humans—a zoonotic disease considered to be rare in the past. Mpox is identical to the Vaccinia virus, the live component of the vaccine against smallpox. The vaccine against smallpox provided cross-protection against other Orthopoxviruses. For instance, it protects against mpox virus with 85% effectiveness [31]. Due to the elimination of smallpox in 1980, routine immunization against smallpox ceased. Hence, no vaccine has been administered against Variola Virus (VARV) for about 40 years, leaving the human population exposed to other Orthopoxviruses [15].

The Democratic Republic of Congo (DRC) is experiencing an mpox epidemic. In January 2023, DRC reported its highest number of mpox cases; a total of 18,922 suspected cases of mpox, including 1007 deaths, as of 29th March 2024 [32]. As of 28th July 2024, there have been a total of 13,791 reported cases of mpox in the Democratic Republic of Congo, resulting in 450 deaths since the beginning of the year [33]. The recent outbreaks in DRC involved urban areas which have the densest population. DRC has a high rate of urbanization of about 47.44% which has been attributed to push factors such as escape from conflict, leading to a rapid decline in the standard of living and the spread of diseases in urban areas [34]. Hence, unlike in previous mpox outbreaks which are confined to the rural and forest regions of the Democratic Republic of Congo, cases are now being reported from urban areas like the capital city of Kinshasa, where the virus does not typically occur [15, 31]. The geographic expansion to previously unaffected areas represents a shift from previous outbreak patterns, where the transmission of mpox virus was mainly limited to rural areas.

Significant changes in the transmission dynamics of mpox have been observed in the recent outbreaks. The means of transmission reported in these cases involved zoonotic, human-to-human close contact, and human-to-human sexual transmission, especially in children and among gays, bisexuals, and men who have sex with men (GBMSM) and female sex workers [15, 35]. Almost all case groups of mpox include an individual between ages 20 and 50 who is GBMSM. However, mpox was not known to be sexually transmitted in the past [31]. The changes in transmission dynamics have been linked to a combination of factors, such as livelihood seeking, transactional and professional sex work, risky occupations like mining, displacement, and conflicts [36]. The epidemiology of mpox is evolving, the different clades have been noted to have different epidemiological patterns. Clade 1 is endemic to Central Africa and can cause severe illness. Initially, it was primarily of zoonotic origin, but there has been an increase in human-to-human transmission. In March 2023, instances of sexual transmission were observed in the Democratic Republic of the Congo [37].

Cross-border movement among neighboring countries has led to the rapid spread of mpox [38]. Countries surrounding DRC have been reporting the incidence of the Clade 1 mpox—a subclass of MXPV endemic to DRC. In late July 2024, there were 213 reported cases in the Central African Republic (CAR) which borders the north of DRC. In addition, Burundi and Rwanda, which border the east of DRC, reported eight cases and two cases of mpox, respectively [33, 39]. On the western border of DRC, the Republic of the Congo (ROC) announced a Clade I mpox outbreak in April 2024, reporting 146 cases. Further west, Cameroon and Nigeria have reported 35 cases and 24 cases, respectively [33]. The pattern of recent outbreaks in African countries reveals that the immediate neighboring countries, especially the west of DRC are the most affected [33]. According to an epidemiological investigation of individuals with MPXV infection reported in Uganda, transmission occurred outside the country. Those with mpox in Kenya were identified at a point of entry and confirmed to be infected with the clade 1b mpox.

### Current drivers of the resurgence

Human mpox virus is caused by a zoonotic orthopoxvirus that resembles smallpox and it is quite difficult to differentiate the illness from the likes of varicella and smallpox. The mpox virus is transferred by direct exposure with respiratory droplets, body fluids, and contaminated objects like blood [40–43].

There are varied socioeconomic factors confirmed to be a major driver of mpox virus in Africa, some of which have been well researched by some scholars in recent times. In an assessment performed in mpox-affected areas of rural the Democratic Republic of Congo, the lifestyles and demographic associated with presumptive risk factors for mpox virus infection was described using a risked questionnaire. Two indices were generated to assess risk: household materials index (HMI), a proxy for socioeconomic status of households and Risk Activity Index (RAI). It was discovered that people in this population are more likely to visit the forest than a market to fulfill material needs, and that reported occupation is limited in describing behavior of individuals who may participate. Being bitten by rodents in the home was commonly reported and this was significantly associated with a low HMI. The highest scoring RAI sub-groups were ‘hunters’ [4, 13, 44–46]. In rural areas in Congo, it is identified that school-aged males were most frequently identified as the first person infected in the household and were the group most frequently affected overall. Risk factors of acquiring mpox virus in a household included sleeping in the same room or bed or using the same plate or cup as the primary case. There is no significant risk associated with eating or processing of wild animals [7, 45–48].

In the past, mpox predominated primarily in the rural rainforests of Central and West Africa. Recently, the exportation of mpox from Africa to other continents has been progressively reported. However, the lack of travel history to Africa in most of the 2022 reported cases promotes the sign of changing epidemiology of this disease. Concerns over the geographic distribution and continued resurgence of mpox are growing. The precise cause of the resurgence in mpox cases is mostly unknown, several suggested factors are believed to be waning immunity, accumulation of unvaccinated people, ecological conditions, risk behaviours of men who have sex with men, and genetic evolution [7, 45, 46, 49].

Studies have shown that there are known environmental drivers which pose a significant influence on the existence and reemergence of mpox. A Bayesian Hierarchical generalized linear mixed model was conducted to identify the associations between mpox incidence and three types of environmental risks factors illustrating the environment as a system resulting from physical, social, and cultural interactions primary forest, economic well-being, and temperature were positively associated with annual mpox incidence [46, 49, 50]. Studies show that physical environment risk factors alone cannot explain the emergence of mpox outbreaks in the the Democratic Republic of Congo [49, 50]. Economic level and cultural practices participate from the environment as a whole and thus, must be considered to understand exposure to mpox risk [51, 52].

There has been a huge challenge and concern about Africa's capacity to diagnose and contain the disease. The challenges faced by Africa include a lack of adequate laboratory infrastructure and health care workers, weak disease surveillance systems, and a lack of mpox knowledge among health care workers and a lack of Mpox knowledge among health care workers and communities. In addition, it has also been said that many popular areas in Africa have health care facilities that do not have refrigerators or reliable power supply to keep the specimens viable until they are transported to centers, where the tests will be performed [53]. There is also an overwhelming shortage of health care workers in Africa, which makes it unimaginably difficult for countries to collect specimens for laboratory confirmation. The tools required to contain the mpox outbreak, such as diagnostic, vaccines, therapeutic, and intense surveillance systems, are not readily available to many African nations [54]. It is noteworthy to emphasize that the African continent is almost not ready for the containment of the mpox virus, as its public health infrastructures are not optimized enough to fight the deadly outbreak. Deficiencies affecting the quality of data collected for surveillance of infectious diseases include capturing incomplete data and the inconsistency of the data at different levels of the surveillance system [48, 54, 55].

### Lessons from recent outbreaks

Mpox disease caused by the mpox virus, from history is endemically linked to the central and western Africa with major outbreaks in countries like Republic of the Congo, Nigeria, Sierra Leone, Benin, the Central African Republic, Cameroon, Gabon, Egypt, Ghana, Liberia, Morocco, Mozambique, Nigeria, Sudan, and South Africa [56]. It was first discovered in Costa Rica (1958) and the first human case was reported in a 9-month-old baby in the Republic of the Congo in 1970 [23, 57]. With recent spread to countries not endemic to the disease and resurgence in the very endemic countries have raised serious public health concerns [58]. As of July 2022, 52 years since the first human case was identified, about 5783 cases were confirmed by the center for disease control and prevention (CDC) in 52 different countries [58, 59]. With all these, there is a need to carefully analyze the factors that may have led to the resurgence and the spread of the disease to enhance prevention and control measures for future outbreaks.

It was, however, identified only in animals [23]. The republic of Congo recorded the first human case of mpox disease was first seen in the Republic of the Congo in 1970. Though mpox is a zoonotic infection, human-to-human transmission of the mpox have been seen since the 1970s in the Republic of the Congo. The disease was initially present in 11 of 26 provinces and in over 54 years, since first case has spread to 22 of 26 provinces in the country with links to neighboring regions. Of the two clades of the mpox virus clade I, previously called the Congo Basin clade; and clade II, previously called the West Africa clade, only the clade I has been identified in DR Congo [60]. The global outbreaks mpox virus infection, only the clade IIb has been linked to sexual interactions particularly amongst men having sex with men, but a recent outbreak in the country as of April 2023 revealed the first cases of clade I mpox virus infection linked with sexual interaction. The first known case was seen in a Belgian man who visited the Republic of the Congo and tested positive for clade I in Kenge, Kwango province, and has had multiple sexual interactions with other men [60]. Several outbreaks have been reported to happen at the same time throughout the country with different features based on each province. Some notable regions with outbreak include Kenge which is about 260 km from the capital city of Kinshasa. Most cases were linked to men who have sexual relations with other men. Another important region is Kinshasa, the first identified case was in August 2023 and four different events were identified, all were travelers from infected provinces. A total of 18 cases were confirmed with a death (other comorbidity present) with a male-to-female sex ratio at 2:1 [60]. Finally, South Kivu, the first ever mpox case was identified in September 2023 from a traveler from an infected province as well. They recorded 34 confirmed cases with a majority being sex workers [60, 61]. The new reports of human-to-human transmission of mpox in areas like Kinshasa indicates that the epidemiology of mpox in the Democratic Republic of the Congo is changing. Also, this recent outbreak is largely linked to men having sexual relations with other men in some provinces, while other provinces through contact with dead or live animals, in-house transmission with high proportion of these case being children less than age 15. Finally, regions like South Kivu with conflict, food crisis, displacement etc., could help increase the spread of mpox [60]. In summary, since the re-emergence, the republic of Congo has the highest reported cases of 4480 from a total population size of 109 million people as of 11th August 2024 with 21 deaths, 0% fatality ratio, 3235 cases in 2024, 19 deaths in 2024, 38 cases in the past 2 weeks with 0 deaths and then predominantly Clade Ia and IIb mpox virus [62]. The following highlights government/public health responses to the recent mpox outbreak in the Democratic Republic of the Congo; a special mpox preparedness budget was created by the minister of public health. An mpox emergency response team was created to cover specific aspects including monitoring and detection, education, communication and awareness, training and capacity building for workers and caregivers [62].

The federal republic of Nigeria reported the first human case of mpox in April of 1971 in a 4-year-old girl in Abia state and subsequently the first human-to-human transmission was seen in the mother of the 4-year-old girl. A third case was also identified in the Oyo state (1978) all without a proper identification of the primary source of infection. There were no reported cases between 1978 and 2017 but a resurgence of the disease started in September of 2017 with a confirmed case of an 11-year-old boy Yenagoa Local Government Area (LGA) in Bayelsa State, who initially presented at the hospital as chickenpox but was later confirmed to be mpox disease [63–66]. The index cluster at that point was in families all of whom had contact with monkeys prior to infection [65]. The recent outbreaks since the 1970s started in 2017 was first identified in Bayelsa state (southern Nigeria) but in the past 7 years have spread across different regions of the country including 32 states and the Federal Capital Territory (FCT) Abuja. The states include Abia, Akwa-Ibom, Anambra, Bayelsa, Cross River, Delta, Edo, Enugu, Imo, Lagos, Nasarawa, Oyo, etc. Abia, Akwa-Ibom, Anambra, Bayelsa, Cross River, Delta, Edo, Enugu, Imo, Lagos, Nasarawa, Oyo, Plateau and Rivers and the Federal Capital Territory (FCT) [65–67]. Primary sources of infection in the earliest index cluster cases were traced to contact with monkeys. This further suggests a primary driver of the current spread of mpox cases in the country and also the increasing interaction of humans with wildlife most especially in the northeast. This interaction largely due to poverty, deforestation, conflict has further led to migration into the forest with increasing contacts with wildlife. Another important driver of the spread can be linked to the poor disease surveillance and monitoring system particularly in the rural areas [67, 68]. Cessation of the smallpox vaccine since1980 and the reduction in herd immunity could have been a factor for the re-emergence even in people initially vaccinated for smallpox as well [67]. Between September 2017 and September 2018, 269 suspected cases, 115 confirmed cases across 16 states and 7 deaths were recorded of which 4 had pre-existing immunocompromised conditions [65]. Of all the cases, two were health care workers among the confirmed cases. The most affected age group is 21–40 years and 79% of the confirmed cases are males, about 8% were reported to have had contact with animals, 9.8% had close contact with a confirmed case, and more than 70% had no known source of exposure [63]. The 12 close contacts of confirmed cases included 1 prison inmate, 12 health care workers who participated in the management of cases and n1ine household contacts, 5 of whom were relatives of the index case [63]. This clearly shows that within a year, it spread from one (1) state (Bayelsa) to sixteen states indicating a poor curb of the disease. This period between 2018 and 2022 saw the exportation of the disease from Nigeria to other countries including the United Kingdom, USA, Israel, and Singapore [63, 69]. Noted cases include 4 individuals traveling from Nigeria to the United Kingdom (2), Singapore (1) and Israel (1), and these were the first human mpox cases exported from Africa [69].

Overall, since the re-emergence in 2017, Nigeria with a population of over 200 million people has a record-high reported cases of 901 as of 18th August 2024 [62]. The 901 reported cases, 9 deaths, 1% fatality ratio, 40 cases in 2024, 0 deaths in 2024, 2 cases in the past 2 weeks with 0 deaths and was predominantly caused by Clade II mpox virus [62]. For intervention, WHO reported that the Federal Ministry of Health through the Nigeria Centre for Disease Control (NCDC) in collaboration with the State’s Ministry of Health are investigating suspected cases and a close monitoring and follow-up on contacts. Modified surveillance is also ongoing in all states especially in the most affected states like Bayelsa, Lagos and in the federal capital territory, Abuja. As an addition, Animal surveillance in collaboration with the United States Centers for Disease Control and Prevention has been put in place [65]. Due to the low number of cases and mortality in other countries outside the democratic republic of Congo, Nigeria, a summary of the incidence of mpox in these countries has been considered and they include: Burundi reported 153 reported cases, 0 death, 0% fatality ratio, 153 cases in 2024, 0 deaths in 2024, 113 cases in the past 2 weeks with 0 deaths and Predominantly Clade Ib mpox virus [62]. For Ghana, there were 127 reported cases, 4 deaths, 3% fatality ratio, 0 cases in 2024, 0 deaths in 2024, 0 cases in the past 2 weeks with 0 deaths and predominantly Clade II mpox virus [62]. The Central African Republic had 92 reported cases, 2 deaths, 2% fatality ratio, 41 cases in 2024, 1 death in 2024, 2 cases in the past 2 weeks with 0 deaths and predominantly Clade Ia mpox virus [62]. Cameroon, 50 reported cases, 5 deaths, 10% fatality ratio, 5 cases in 2024, 2 deaths in 2024, 0 cases in the past 2 weeks with 0 deaths, and predominantly Clade Ia and II mpox virus [62]. Congo reported 45 cases, 2 deaths, 4% fatality ratio, 19 cases in 2024, 0 deaths in 2024, 0 cases in the past 2 weeks with 0 deaths and predominantly Clade Ia mpox virus [62]. South Africa had 29 reported cases, 3 deaths, 10% fatality ratio, 24 cases in 2024, 3 deaths in 2024, 0 cases in the past 2 weeks with 0 deaths and predominantly Clade II mpox virus [62].

It is seen that increased interaction of humans with wildlife have further increased the spread of the mpox virus. This interaction with wildlife is largely due to poverty, conflict and migration of human into wildlife. It is also learnt that poor disease surveillance and limited monitoring system in rural areas are also very important driving factors for the mpox disease [67]. In many African regions, it is seen that there is massive underreporting which is still linked to the poor surveillance and limited diagnostics, in the long run altering the available data and outcome of the management of present and future outbreaks [70]. Another lesson of note is the poor power supply to in many African health facilities to keep specimens viable till they are transported to the necessary centres for testing [53]. From the epidemiological trends, it was also learnt that both pharmacological (the use of vaccines and non-pharmacological approaches were pivotal in the control of the virus. These non-pharmacological approaches are the social distancing, proper handwashing routine, use of proper personal protective equipment (PPE) have proven vital in preventing the spread of mpox virus, largely because the human–human transmission is largely the contact with fluids of all sorts from the infected person [4, 5]. A vital lesson worthy of note; would be providing emotional and psychological support for health workers as the outbreaks took a toll on the mental health of health workers [71]. In all these, it is important to explore, maximize and modify the lessons learnt from these outbreaks so as to give proper orientation to health workers so they are better equipped to handle future outbreaks [4, 6]. Strategies to employ includes increasing awareness and knowledge on the mpox communities amongst locals and as well as health workers to improve case management and prevention of the mpox disease. In addition, providing sufficient PPEs to health workers. Vaccination should also be largely employed particularly in countries who have experienced outbreaks to minimize the effects of the mpox virus [42, 71].

### Successes and failures in mpox outbreak response in African contexts

The mpox is one of the numerous public health crises faced by Africa as a continent. We have seen diseases like HIV/AIDS, Ebola virus disease, COVID-19 and other infectious diseases grapple the continent in the past and it is a reminder of the important and critical role of an effective public health response in curbing the spread of infectious diseases [67]. While some African countries have recorded massive improvement and successes in minimizing the spread of the mpox, others have a long way to go as they are facing significant challenges. We will review some of the factors that have contributed to both the successes and failures of these response efforts, to identify lessons helpful for future outbreaks. In terms of Diagnostics capacities, the COVID-19 pandemic was an eye opener and a plus in the reinforcement of laboratory capacities. All African countries are in possession of PCR machines needed to test for mpox. A bit of improvement in this aspect as compared to previous outbreaks [70]. Coordination and Support from WHO and Africa CDC made a world of difference as WHO African Regional Office (AFRO) did a review of the incidence management team, this brought about an expansion in that regard. After a meeting in South Africa, an establishment of a joint mpox task force between WHO and Africa CDC has been a significant success, which promoted coordinated efforts to manage the outbreak [28]. There also seems to be an improvement through Risk Communication and Community Engagement (RCCE). WHO has produced and broadcast interactive programs in multiple regions and languages, conducted public sensitization, and engaged in home visits and advocacy with political authorities to raise awareness and manage the outbreak. Through the RCCE, an initiative explored in trials in certain parts of Congo like Sud-Kivu. It involved the use tools like DIGIMIND that collected discussions and rumors about mpox. This helped make informed decisions going forward. Some countries had Laboratory upgrades as well. Countries like South Africa and Liberia have benefited from the support of WHO in procuring reagents to improve logistics of specimen collection and even transportation which overall ensures timely delivery across all health facilities. In managing cases, the distribution of tecovirimat, a mpox therapeutics by WHO to South Africa, participation in clinical webinars as well has led to an improvement in mpox management. Home-based care guidelines for mild cases have also been employed to enhance regional preparedness [39]. Based on presentable reports, there was relative improvement in Surveillance and case reporting. With the help of WHO, there is an advancement in the upgrading of surveillance infrastructures, particularly community-based surveillance programs in places like the Internally Displaced People’s (IDP) camps that pose high risks. A country like Nigeria has utilized the use of digital tools and case reporting which is an advancement and overall improved the management of the disease [39]. Responses to disease outbreaks got better as well. South Africa for example has undergone sub-national readiness reviews, this helped focus on non-infected provinces. This helped to properly prepare for a potential outbreak in those provinces. WHO has provided support as well to different countries to increase their readiness capacity [39].

With all the mentioned above, there has been relative improvement in overall management and in curbing the disease in Africa as compared to previous outbreaks and positioned the public health teams better in case of another endemic outbreaks. However, there is still a long way to go particularly in the rural areas as several factors have led to significant lapses in the management of the disease. As much as successes were recorded in certain countries, an overview of the situation shows that Africa as a continent has a long way to go. Studies from different articles, since the resurgence of the mpox disease has identified numerous challenges which have led to the failure in the management of the disease. The African countries most affected by the mpox disease are either developing or underdeveloped and this translates to inadequacies in the advanced management of the viral disease. Nigeria, the largest economy of Africa lacks the access to high-quality equipment, such as MRI scanners and oxygen tanks, as well as well-equipped laboratory facilities, inside hospital settings of which are vital in combating a viral infection like the mpox [67]. In addition, this is the fate of most of the other African countries as they fall in the developing pool. Some of the other factors and failures identified include areas of diagnostics, although mpox confirmatory tests are available worldwide, Africa as a continent has struggled in this regard. Challenges faced by Africa include the lack of sufficient laboratory infrastructure and even in cases, where infrastructures are intact, there is shortage of manpower with the know-how to operate them [16]. Poor surveillance systems and delayed response is also a notable barrier, and this is largely due to limited resources and particularly absence of health workers with adequate mpox knowledge [39, 67, 70]. In many African countries, the lack of resources has caused delay in responses and overall altering the curb of the disease. There is massive under-reporting in many African regions due Limited surveillance and insufficient diagnostics, and this alter the available data and overall, the outcome of the management of the present and future outbreaks [70]. Limited resources in Countries like Burundi and Cote d’Ivoire have created significant difficulties surveillance and diagnostics [39]. Also noted is the high level of ignorance and stigmatization due to lack of proper information of the mpox, individuals and communities have promoted stigmatization of affected people. This also contributed to the failure in responses [39]. The unending delay in approval and regulation processes have caused a limitation of access and distribution of antiviral treatments like tecomirat have further complicated the situation. The lack of awareness and training has hindered effective identification, reporting, and management of cases. This is worsened in countries with no prior incidence of the mpox [39].

### Impact of vaccination and treatment strategies in African settings

Vaccination is an essential part of disease management. To a significant extent, the vaccine for smallpox gives immunity to the mpox, but just as every other continent, the halt of smallpox vaccination campaigns worldwide due to the eradication of the disease may have made certain age group more susceptible to mpox [72]. There also is scarcity and a heavy limitation in the distribution of mpox vaccines especially to rural areas with high numbers of cases. This has significantly reduced the joint effort to the eradication of in Africa [72–74]. Vaccination for health workers who directly treat infected patients and certain individuals who are highly exposed to animal reservoir species could be considered going forward. Vaccines such as the modified vaccinia Ankara or the LC16M8, which have the potential to bypass problems associated with the current smallpox vaccine should be strongly considered since the unavailability of the monkey pox vaccine in Africa [74, 75]. For example, Nigeria, like most African countries affected, has yet to receive a dose of mpox vaccines, not even for clinical trials [63].

Largely, treatment of mpox has primarily been based on managing clinical symptoms, this includes managing skin care, reducing the pain, preventing, and managing complications as well. In regions where availability is enhanced through emergency or certain programs, specific antiviral medications such as tecovirimat have been used in the treatment of mpox, mostly in patients with severity or high risk [39]. Based on region, three vaccines are available, they are MVA-BN, LC16-KMB, and OrthopoxVac—yet to be commercialized. It is recommended by WHO to use MVA-BN or LC16 vaccines when others are unavailable [39]. The importance of vaccination in mpox as to every other infectious disease cannot be overemphasized. Vaccination is strongly recommended by WHO for individuals at high risk of exposure [39]. For a positive improvement in curbing the spread of mpox, there is a strong need for Africa as a country to invest in distribution of vaccines for maximum effectiveness in the management of the disease.

## Future directions and recommendations

Countries within Africa with marked endemicity should adopt surveillance systems that outperform conventional surveillance systems which are paper based and manually transferred [76]. The Surveillance Outbreak Response Management and Analysis System (SORMAS) is a digital system that structures and enables infectious disease control and outbreak management procedures, as well as disease surveillance and epidemiologic analysis at all levels of a public health system [77]. In Nigeria, the Outbreak Response Management and Analysis System (SORMAS) or mpox surveillance across portions of eight states was implemented for the mpox outbreak [78]. The use of the system increased the quantity of epidemiological data collected and the communication of aggregate case data [69].

Person-centered qualitative research will be important in sharing insights about the mpox sequelae at the individual and community levels that would otherwise have been missed by epidemiological and clinical studies. To achieve these goals, strong multisectoral advocacy for funding and policy support is essential, as shown during the high-level emergency regional meeting on mpox in Africa, in April, 2024 [79]. Strengthening health care systems is also important. Enhancing laboratory capacity for early diagnosis and confirmation improves response times, as Precious et al. emphasize [80]. In addition, increasing infrastructure, human resources, and funding for outbreak response equips health care systems to handle future challenges, as highlighted by Moyo et al. [70]. Molecular diagnostic tools, such as polymerase chain reaction assays, play a vital role in swiftly confirming mpox cases [81]. Sentinel surveillance sites positioned in endemic regions within Africa aid the monitoring of febrile illnesses, enabling rapid identification of potential mpox cases. In addition, syndromic surveillance, focusing on specific clinical symptoms associated with mpox, aids in the early recognition of outbreaks [57]. The integration of data from animal health surveillance systems, emphasizing potential zoonotic spillover events, further enhances the sensitivity of surveillance networks. Although much attention is given to human–human contact tracing, great efforts need to be put into animal–animal and animal–human contact tracing especially due to non-specificity of reservoir hosts for mpox virus [82].

The inadequate data regarding the epidemiology of mpox virus highlights the importance and advantages of doing research in any given nation. The study of the virus enables researchers and professionals to gain a more comprehensive grasp of its characteristics, including its modes of transmission and its genetic variants. Current research focuses on refining and augmenting existing smallpox vaccination strategies, given their partial cross-protection. Novel vaccine candidates, such as the live attenuated modified vaccinia ankara vector expressing mpox antigens, have shown promising results in preclinical studies, eliciting robust immune responses, and conferring protection against virulent mpox challenge [83]. Based on the available data, there remains a need to advance the development of effective and secure novel vaccines designed explicitly for mpox. This necessitates exploring innovative vaccine approaches, including virus-like particles, recombinant protein, nucleic acid (mRNA or DNA), and nanoparticle-based vaccines [84]. In addition, there is a need for novel biotechnology studies on the mpox virus to spur the production of low-cost treatments and vaccines. Healthcare professionals also have the option to employ a technique known as “ring-vaccination” as an alternative approach to immunize individuals who have had close contact with confirmed cases of mpox. This strategy aims to disrupt transmission chains and effectively confine the spread of the virus as highlighted by Ogunkola et al. [85]. In low resource settings, vaccination exercises should be targeted at vulnerable populations. Hunting, handling, preparing and consuming bushmeat was consistently implicated in developing clinical mpox in endemic African countries [86]. Focus on strengthening outbreak teams to investigate index case interaction with wildlife will help to build local knowledge. West and Central Africa face diagnostic challenges associated with scarce access to nucleic acid amplification testing platforms and cold chain requirements for sample preservation, which hamper case confirmation in remote areas and, therefore, an understanding of mpox epidemiology. In rural health clinics or regional hospitals in low-income countries without access to high-precision polymerase chain reaction instruments, loop-mediated isothermal amplification diagnostic assays could be a viable alternative. Coordination and communication between human and veterinary health services should be improved within and among countries to ensure that animal outbreaks are quickly communicated to health care personnel who will institute prevention measures needed to prevent the spread of the disease to human. There should also be a collaboration of ministries of health of different countries. African governments must establish an intercountry early warning system for infectious disease outbreaks. Communities must be warned about impending outbreaks early so that they can prepare and take preventive measures to reduce morbidity and mortality. There should be a communication of risk messages to communities’ at large and specific populations at high risk, such as sex workers, immunocompromised individuals. Messages that should be emphasized include reducing human contact with suspect animals, avoiding physical contact with infected individuals, frequent handwashing, and early medical examination of suspected cases. Measures to improve infection prevention and control include contact precautions, appropriate disinfection, and limited contact with patients for healthcare practitioners are also vital.

To prepare for future outbreaks, early notification of anticipated outbreaks is necessary to enable communities to prepare and take preventive measures, which will lower morbidity and mortality [87]. Risk messaging should be disseminated to the public and high-risk groups, such as minors, immunocompromised individuals, and sex workers [54]. Population at increased risk for contracting mpox is primarily those who have close physical contact with symptomatic mpox-infected individuals. In non-endemic regions, a large proportion was identified among lesbian, gay, bisexual and queries (LGBTQ), particularly men having sex with men (MSM) [88]. To avoid stigma, it is important that monkey pox is not labelled as a sexually transmitted disease. Mpox being mis-portrayed as a sexually-transmitted infection fails to recognize others at risk, spurs stigma and interferes with implementation of precautionary measures, access to healthcare services. Therefore, more effective measures to destigmatize mpox should be developed and implemented. The destigmatization of human immunodeficiency virus infection (HIV) lends strategies to combating stigma and enhancing community engagement in relation to mpox [89]. Identifying the transmission mode is helpful in reducing stigma and health inequality. Previous evidence has found that the primary transmission of mpox virus could be through broken skin, respiratory tracts, eyes, noses, or mouths (animal or human) [42]. In secondary transmission, aerosols play an important role. Therefore, it is not appropriate to draw a direct link between mpox infection and homosexuality. Equally, information on mpox should be disseminated properly and accurately. The terminologies and descriptions used to describe mpox are crucial in the establishment of its stigma. In the 1980s, media described HIV as “gay-related immunodeficiency” and “gay cancer”, which enhanced HIV stigma globally for several decades. Identifying and disseminating mpox information properly and accurately can remove social perceptions about mpox being caused solely by sexual misbehavior. Community engagement should be strengthened to decrease the proliferation of stigma. Gatherings and events, where physical contact may be prolonged including intimate or sexual contact may represent conducive environments for the transmission of mpox. Event organizers, religious authorities and other stakeholders must be aware of preventive measures and adhere to public health guidelines by the World Health Organization [90]. The change in transmission dynamics with increased human-to-human transmission bears public health implications. Public health experts, regional health authorities, and gay, bisexual, and other men who have sex with men (GBMSM) communities must work together in concert to face this situation. These may involve deploying education campaigns and vaccination opportunities targeting high risk groups including partner-seeking platforms and digital networks that contribute to sexual activity with multiple partners [91]. Beyond community-based organizations that serve the community, public health providers must equally establish robust partnerships with sex-on-premises venues that cater to GBMSM. Key public health messaging should link mpox to other public health needs and envisage beyond intimacy related transmission. Risk communication should equally acknowledge all routes of mpox transmission and utilise media campaigns that go beyond endemic African countries and GBMSM communities to avoid discrimination and stereotype. Primary care providers, emergency care centres, sexual health clinics and other health facilities must be aware of communication guidelines that will help curb stigma and refrain from panic inducing messages.

## Conclusion

This study highlights the resurgence of mpox as a significant public health challenge, driven by a combination of socio-economic, environmental, and epidemiological factors. The resurgence is closely linked to waning immunity due to the cessation of smallpox vaccination, increased human–wildlife interactions, and the rapid urbanization of endemic regions. The spread of the virus into urban areas and across borders, exacerbated by high population mobility and socio-economic instability, has shifted the transmission dynamics, with recent outbreaks increasingly associated with sexual contact, particularly among men who have sex with men (MSM). These factors, coupled with the genetic evolution of the virus, have resulted in a broader and more virulent outbreak, emphasizing the need for robust surveillance and targeted public health interventions. The lessons learned from these recent outbreaks establishes the critical importance of strengthening health systems across Africa, particularly in terms of diagnostic capacity, surveillance, and response readiness. The challenges faced, such as inadequate laboratory infrastructure, insufficient healthcare worker training, and delayed response mechanisms, have highlighted the vulnerabilities in the continent’s public health framework. Moving forward, it is essential to implement comprehensive strategies that include enhancing community engagement, improving access to vaccines and treatments, and ensuring timely and accurate reporting of cases. These efforts must be supported by coordinated global action to prevent future outbreaks and mitigate the risk of mpox becoming a more widespread and persistent public health threat.

## Data Availability

Research study did not use data.
